# Impact of CFTR modulation with Ivacaftor on Gut Microbiota and Intestinal Inflammation

**DOI:** 10.1038/s41598-018-36364-6

**Published:** 2018-12-13

**Authors:** Chee Y. Ooi, Saad A. Syed, Laura Rossi, Millie Garg, Bronwen Needham, Julie Avolio, Kelsey Young, Michael G. Surette, Tanja Gonska

**Affiliations:** 1 School of Women’s and Children’s Health, Medicine, The University of New South Wales, Sydney, NSW Australia; 2 Molecular and Integrative Cystic Fibrosis (miCF) Research Centre, Sydney Children’s Hospital, Randwick, NSW Australia; 3 Department of Gastroenterology, Sydney Children’s Hospital, Randwick, NSW Australia; 4 Department of Medicine, McMaster University, Hamilton, Ontario, Canada; 5 Department of Biochemistry & Biomedical Sciences, McMaster University, Hamilton, ON Canada; 6 Farncombe Family Digestive Health Research Institute, McMaster University, Hamilton, ON Canada; 7 Department of Paediatrics, Division of Gastroenterology, Hepatology and Nutrition, The Hospital for Sick Children, Toronto, Ontario, Canada; 8 Translational Medicine, Research Institute, The Hospital for Sick Children, Toronto, Ontario, Canada

## Abstract

Cystic fibrosis (CF) is caused by mutations in the cystic fibrosis transmembrane conductance regulator (CFTR) gene. Next to progressive airway disease, CF is also associated with intestinal inflammation and dysbiosis. Ivacaftor, a CFTR potentiator, has improved pulmonary and nutritional status but its effects on the intestinal microbiota and inflammation are unclear. Hence, we assessed the changes on the intestinal microbial communities (16S rRNA variable 3 gene region) and inflammatory markers (calprotectin and M2-pyruvate kinase [M2-PK]) in 16 CF individuals (8 children and 8 adults) before and after (median 6.1 months) ivacaftor. Stool calprotectin significantly decreased following ivacaftor (median [IQR]: 154.4 [102.1–284.2] vs. 87.5 [19.5–190.2] mg/kg, P = 0.03). There was a significant increase in *Akkermansia* with ivacaftor. Increased abundance of *Akkermansia* was associated with normal stool M2-PK concentrations, and decreased abundances of *Enterobacteriaceae* correlated with decreased stool calprotectin concentrations. In summary, changes in the gut microbiome and decrease in intestinal inflammation was associated with Ivacaftor treatment among individuals with CF carrying at least one gating CFTR mutation. Thus, CFTR-modifying therapy may adequately improve the aberrant pathophysiology and milieu of the CF gut to favor a more healthy microbiota, which in turn reduces intestinal inflammation.

## Introduction

Cystic fibrosis (CF) is a life-shortening autosomal recessive disorder associated with mutations in the gene coding for the cystic fibrosis transmembrane conductance regulator (CFTR) protein^1^. The CFTR protein plays an important role in epithelial fluid secretion and intra-luminal hydration due to its function as an anion-selective ion channel (mainly chloride and bicarbonate). Defective CFTR leads to accumulation and plugging of inspissated, slow-to-clear mucus on the apical surfaces of epithelial cells in the airway and intestine^2^. From a microbial perspective, this change in the luminal milieu results in ideal conditions for colonization with opportunistic pathogens. Progressive pulmonary failure secondary to microbial colonization, infection and destructive inflammatory response are hallmarks of CF lung disease^2^. Similarly, the gastrointestinal tract is affected by significant alterations in the diversity and composition of microbiota in CF patients when compared to healthy controls^3–5^. This may, in turn, further impact on the host as the gut microbiome plays an important role in host immunology and metabolic capacity^6,7^. Changes in the CF gut microbiome have therefore been speculated to contribute to the development of intestinal inflammation, gastrointestinal malignancy, liver cirrhosis as well as airway colonization in CF^3,8–17^. In regards to intestinal inflammation, both stool calprotectin and M2-PK have been previously reported to be elevated in patients with CF compared to healthy controls^4,9,13,15,16,18^.

Therapeutics in CF have entered an exciting era with the availability of personalized small molecule therapies that target the basic defect(s) in the CFTR protein^19^. Ivacaftor, a CFTR potentiator for CF patients carrying gating mutations such as G551D, has demonstrated impressive pulmonary outcomes, including reduction in pulmonary exacerbations, and improved lung function^20–22^. In the gastrointestinal tract, ivacaftor has been reported to improve the abnormal small intestinal pH and histopathologic changes of inspissated mucin within small intestinal crypts seen in CF^23,24^. In addition, ivacaftor was associated with substantial weight increase, which has been speculated to be related to improvements in gastrointestinal physiology rather than solely by improved pulmonary function^10,18,23–25^. However, the impact of such therapies on the gut microbiome and intestinal inflammation as biological markers of improved intestinal physiology in CF remains unclear.

We hypothesize that improvements in gastrointestinal physiology following commencement of ivacaftor therapy, in patients with CF carrying at least one gating *CFTR* mutation, is associated with changes in the gut microbiome and inflammation. In this study, we examined the gut microbial communities and gut inflammation (using two biomarkers of inflammation – calprotectin and M2-pyruvate kinase [M2-PK]) in children and adults with CF before and after commencement of ivacaftor. As secondary aim, we evaluated the relationships between microbial profiles and gut inflammatory markers calprotectin and M2-PK.

## Results

### Subject characteristics

Stool samples were collected from 16 patients (8 children and 8 adults; 10 females and 6 males) with CF at baseline and after commencement of ivacaftor therapy. There were 14 pancreatic insufficient and 2 pancreatic sufficient patients. The genotype and exocrine pancreatic phenotype of these CF patients are summarized in Table 1.

**Table 1:** Subject demographics and clinical characteristics, including changes in pulmonary function, sweat chloride, faecal calprotectin and M2-PK levels following use of ivacaftor.

Age	Sex	Race/Ethnic	Site	Allele 1	Allele 2	EPF	Δ FEV_1pp_	Δ SC (mmol/L)	Δ Calprotectin (mg/kg)	Δ M2-PK (mg/kg)
5	F	White/Hispanic	S	G551D	DF508	PI	24	−46	−25	−0.5
5	F	White	S	G551D	DF508	PI	12	−5	212	196.5
6	F	White	T	G551D	DF508	PI	1	−38	−32	−2.4
8	F	White	S	G551D	Q220X	PI	23	−76	−46	−4.2
8	M	White	S	G551D	DF508	PI	N/A	N/A	−104	−5
9	M	White	T	G551D	DF508	PI	−2	−30	−205	−886.2
12	F	White	T	G551D	DF508	PI	10	−46	−333	−2.6
15	F	White	S	G551D	DF508	PI	0	−61	−149	8.1
19	M	White	T	G551D	DF508	PI	32	−31	−33	196.5
22	F	White	T	G178R	DF508	PI	24	−51	−367	−2.4
23	M	White	T	G551D	DF508	PI	N/A	−35	−631	−13.9
32	M	White	T	G551D	E585X	PI	19	−4	208	−4.2
33	F	White	T	G551D	DF508	PI	24	−66	−92	−5
40	M	White	T	G551D	Unknown	PS	9	−33	−16	−886.2
44	F	White	T	G551D	DF508	PI	16	−49	1	−2.6
50	F	White	T	G551D	Unknown	PS	10	−31	−168	8.1

EPF = exocrine pancreatic function; F = females; M = males; N/A = not available; PI = pancreatic insufficient; PS = pancreatic sufficient; S = Sydney, Australia; T = Toronto, Canada; Δ Calprotectin = change in stool calprotectin levels following ivacaftor; Δ FEV_1pp_ = change in percent predicted forced expiratory volume in 1 second following ivacaftor; Δ M2-PK = change in stool M2-pyruvate kinase levels following ivacaftor; Δ SC = change in sweat chloride following ivacaftor.

Overall, the median [IQR] age of participants was 17.1 [7.8–32.7] years. Median [IQR] age at time of baseline sample collection of pediatric patients was 7.8 [6.2–11.5] years and median [IQR] age at first sample of adult patients was 32.3 [21.8–42.9] years. Stools post-commencement of ivacaftor treatment were collected at a median [IQR] of 6.1 [5.6–8.2] months.

### Clinical characteristics

The pediatric patients showed an increase in weight z-score from baseline to the time of repeat stool collection while on ivacaftor therapy (median [IQR]: 0.32 [−1.89–0.84] vs. 0.66 [−0.87–1.43]; P = 0.012). There was no significant change in height z-scores in the pediatric population (median [IQR]: 0.39 [−1.53–0.80] vs. 0.11 [−1.58–0.89; P = 0.89].

In adult patients, there was a significant increase in body mass index (median [IQR]: 22.4 [21.2–28.2] vs. 23.9 [22.2–28.9] kg/m;^2^ P = 0.028) following commencement of ivacaftor. Furthermore, there was a significant increase in weight in these patients (median [IQR]: 64.1 [54.4–86.7] vs. 65.9 [59.0–88.0] kg; P = 0.028).

There was a significant elevation in the percent predicted forced expiratory volume in 1 second (FEV_1pp_) from baseline to time of repeat stool collection while on ivacaftor (median [IQR]: 76.0 [56.3–96.0] vs. 90.5 [72.5–108.5]; P = 0.002). Sweat chloride concentrations significantly reduced after starting ivacaftor therapy (median [IQR]: 86 [77–91] vs. 39 [36–53]; P = 0.001).

### Stool calprotectin and M2-PK

**Figure 1:**
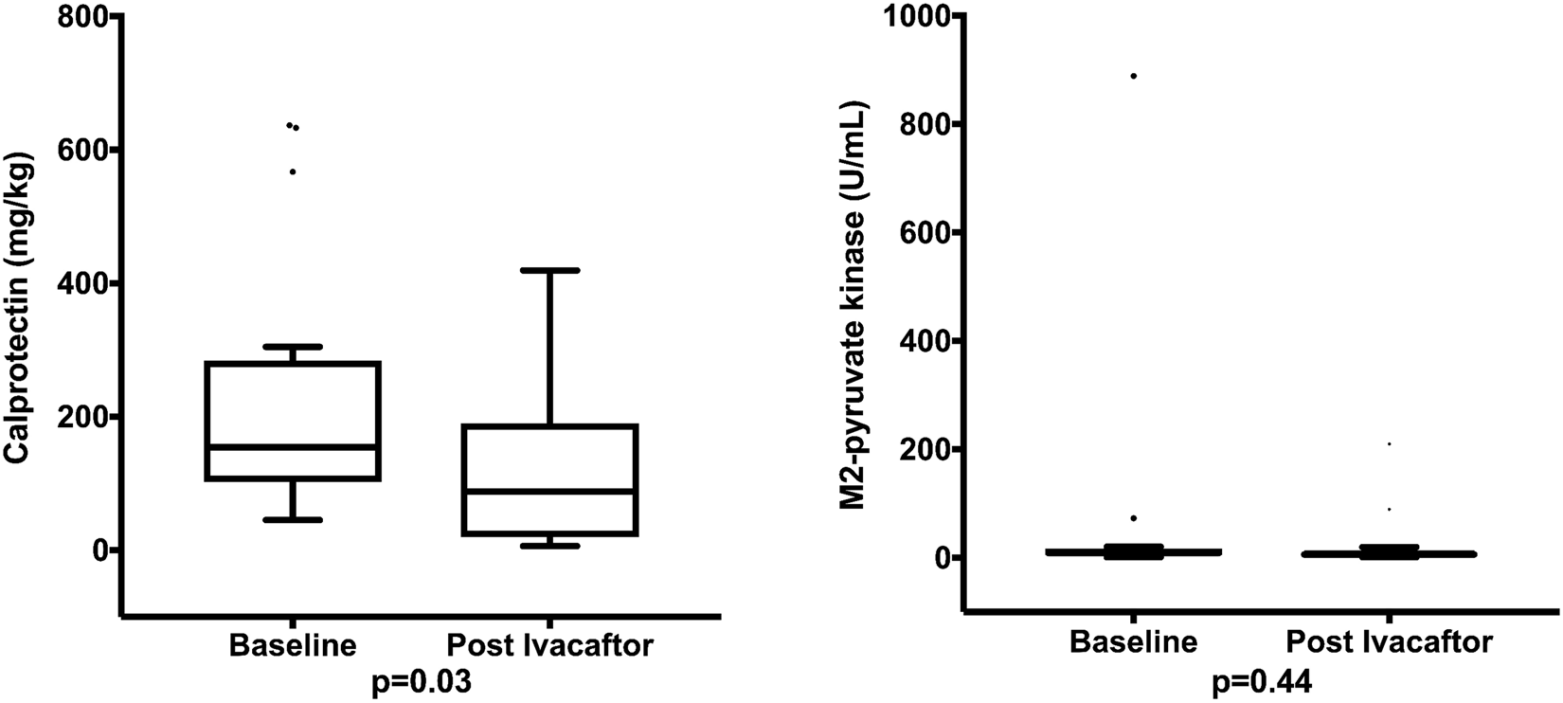
Comparison of fecal (**A**) calprotectin and (**B**) M2-PK before and after ivacaftor. Data are summarized as whisker-box plots presenting median (with interquartile range and range).

There was a significant reduction in stool calprotectin levels after commencement of ivacaftor (median [IQR]: 154.4 [102.1–284.2] vs. 87.5 [19.5–190.2] mg/kg, P = 0.03) (Fig. 1A). In contrast, there was no significant change in stool M2-PK levels from before to after commencement of treatment (median [IQR]: 8.4 [4.8–17.1] vs. 6.2 [2.8–13.3] U/mL, P = 0.44) (Fig. 1B).

### Bacterial microbiome before and after CFTR modulation

**Figure 2:**
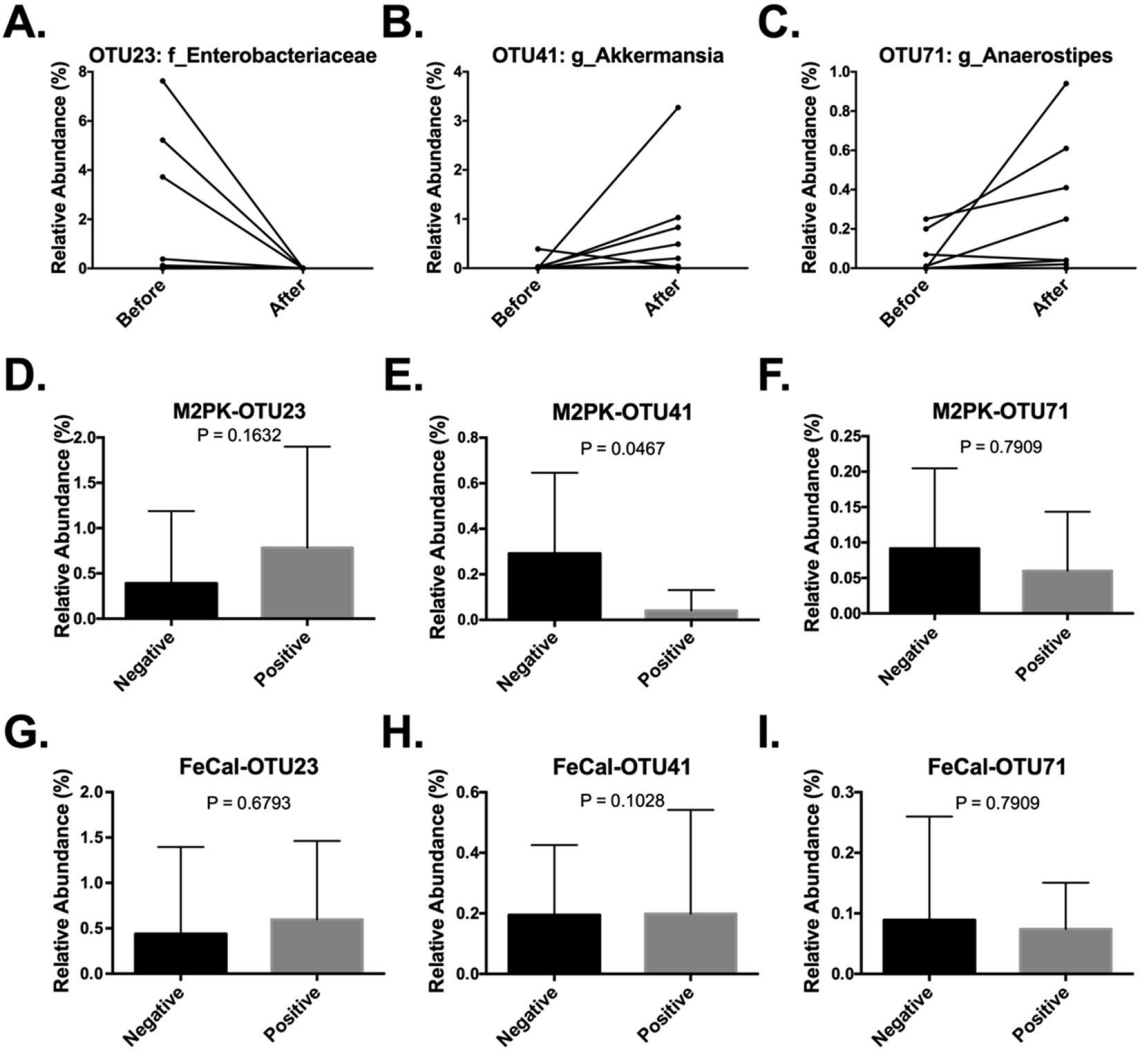
Altered levels of *Enterobacteriaceae* (OTU23), *Akkermansia* (OTU41), and *Anaerostipes* (OTU71) following ivacaftor therapy. (**A–C**) All operational taxonomic units (OTUs) with greater than 10 reads were analyzed for significant differences following ivacaftor therapy using ANCOM. (**D–I**) Relative abundances of relevant OTUs were compared according to categorically negative (normal) or positive (abnormal) stool levels of M2PK and calprotectin for all stool samples (combined before and after ivacaftor). Normal values for faecal calprotectin and M2-PK were based on cut-offs of ≤50 mg/kg and ≤9U/ml. Means and 95% confidence intervals of each OTU’s relative abundance are shown.

Stool samples from before and after treatment with ivacaftor were processed for microbiome profiling using 16S rRNA variable 3 (v3) gene region amplicon sequencing. To test whether any members of the gastrointestinal microbiota were correlated with improved gut health, we conducted Analysis of Composition of Microbiomes^26^. Following treatment, one operational taxonomic unit (OTU) of the family *Enterobacteriaceae* decreased while two OTUs of the genera *Akkermansia* and *Anaerostipes* increased (Fig. 2A–C). After adjusting for the false discovery rate, only *Akkermansia* was significantly increased following commencement of therapy (Fig. 2B).

Stool bacterial microbiome were not significantly different in β-diversity and α-diversity after CFTR modulation, or by sex (Supplemental Fig. S1). However, they were statistically different (R^2^ = 0.09945, P = 0.00148) by location of the clinical centers (Supplemental Fig. S2).

### Bacterial microbiome – correlation analyses in all stool samples

**Figure 3:**
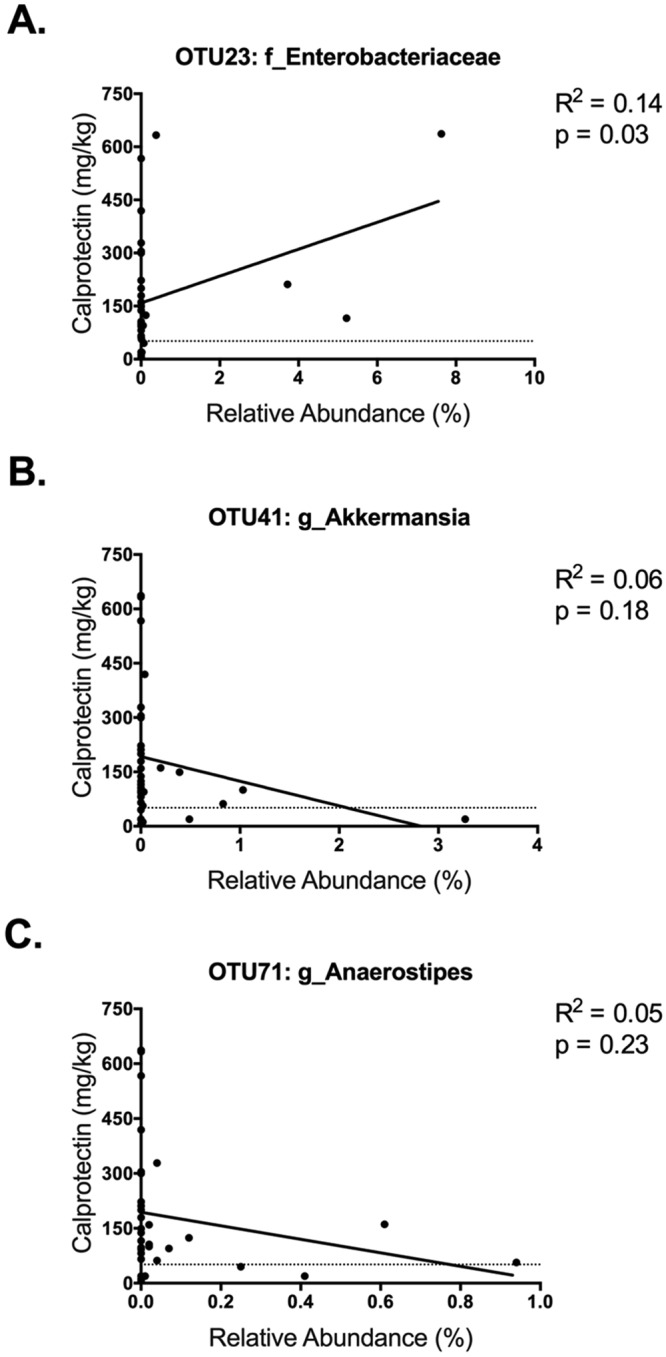
Correlations between stool calprotectin and relative abundances of (**A**) *Enterobacteriaceae* (OTU23), (**B**) *Akkermansia* (OTU41), and (**C**) *Anaerostipes* (OTU71).

Bacterial relative abundances were compared according to categorically negative (normal) or positive (abnormal) stool levels of M2PK and calprotectin for all stool samples. A higher abundance of *Akkermansia* was significantly associated with negative stool M2-PK levels (M2-PK <9.1U/ml) (Fig. 2E). There were no associations between the relative abundances of *Enterobacteriaceae* and *Anaerostipes* with M2-PK levels (Fig. 2D,F). There were also no association between relative bacterial abundances and negative or positive calprotectin levels (Fig. 2G–I). However, on linear regression analysis, there was a significant association between stool calprotectin levels and the family *Enterobacteriaceae*; decreased relative abundances of *Enterobacteriaceae* was associated with reduced levels of stool calprotectin (Fig. 3A). There was no association between stool calprotectin levels and *Akkermansia* and *Anaerostipes* (Fig. 3B,C).

## Discussion

To our knowledge, this is the first study to demonstrate that treatment with ivacaftor improves intestinal inflammation in individuals with CF carrying at least one gating *CFTR* mutation. In this prospective observational study, we also found significant correlation between stool calprotectin levels and *Enterobacteriaceae* abundance, and that treatment with ivacaftor is associated with an increase in the relative abundance of *Akkermansia*.

Many studies have previously reported the presence of intestinal inflammation in CF patients, particularly with significant elevations in stool calprotectin and M2-PK levels in patients with CF compared to healthy controls^4,9,13,15,16,18^. The underlying pathogenesis of intestinal inflammation in CF remain unclear but speculated to be related to physico-chemical changes in the intestinal milieu, which is primarily due to the dysfunction in CFTR^3,11,12,27^, that may in turn favor alterations in the gut microbial community. In this observational study, ivacaftor therapy led to a reduction in stool calprotectin suggesting that the intestinal inflammation may be improved or reversible in patients with CF upon restoration of the intestinal millieu.

With the substantial increase in CF patients surviving into adulthood, the number of patients being diagnosed with gastrointestinal malignancies has increased^17^. The underlying pathogenic mechanisms remain unclear but chronic gastrointestinal inflammation in CF, which begins in early childhood^16^, has been shown to promote oncogenesis^28,29^. The impact of novel CFTR modulator therapies on the incidence of gastrointestinal cancers in patients with CF if of great interest and will require future studies.

The altered microbiome in the CF gut is characterized by a high abundance of *Enterobacteriaceae*, particularly *Escherichia coli*, which has been shown to be 10 times higher in CF compared to healthy controls^8^. Similarly, the *Enterobacteriaceae* family have also been reported to dominate the gut microbial profile in CFTR−/− mice^30^. Ferrets models of CF demonstrated intestinal overgrowth of *E. coli* at 2.5–7.5 times higher compared to controls^31^. Following treatment with ivacaftor, we have demonstrated a weak effect of decreased abundance of the *Enterobacteriaceae* family. We speculate that a statistically significant effect was not observed following false discovery rate adjustment as the *Enterobacteriaceae* family not only includes the familiar and well-established Gram-negative pathogens such as *E. coli* but also many harmless symbionts. We nevertheless propose that there was selective reduction in abundances of pathogenic members of the *Enterobacteriaceae* family (and thus sparing the non-pathogenic symbionts) following ivacaftor therapy due to a positive correlation between stool calprotectin levels and the abundances of *Enterobacteriaceae* in our cohort. This finding is consistent with a previous study reporting significant association between stool calprotectin and *E. coli* abundance^8^. This observation further supports the close link between alterations in the gut bacterial microbiome and the development of intestinal inflammation in patients with CF. Lastly, we demonstrate that modulation of intestinal CFTR function with ivacaftor and thus modification of the intestinal luminal milieu^23^, reverses some of the dysbiosis and inflammation in CF patients.

In our study cohort, members of the bacterial genus *Akkermansia* were significantly increased during therapy. *Akkermansia* is a Gram-negative, mucin-degrading bacteria that resides in the intestinal mucus layer^32^. There has been emerging interest in *Akkermansia* due to the association between its abundance with a healthy gut mucosa. In previous studies, the abundance of *Akkermansia* has been inversely correlated with intestinal inflammatory conditions, such as inflammatory bowel disease, microscopic colitis and appendicitis^33–36^. While its protective mechanisms remain to be fully elucidated, *Akkermansia* reportedly stimulates host mucosal anti-inflammatory pathways, and improves epithelial barrier integrity^37–41^. In addition, *Akkermansia* increases the expression of RegIII, an anti-microbial peptide with direct bactericidal activity against Gram-positive bacteria in the intestine^40^. In our study the increased abundance of *Akkermansia*, which is a biomarker of gut health, was significantly correlated with normal levels of stool M2-PK^42^. The underlying mechanism(s) for increased abundance of *Akkermansia* in CF following ivacaftor therapy is unknown but we speculate that this may be related to the improvement of the characteristics of the naïve CF intestinal mucus. More specifically, improved bicarbonate secretion by CFTR following ivacaftor use may have created a more amenable environment for mucin degraders such as *Akkermansia*, due to improved unfolding and maturation of intestinal mucins^43,44^.

It is notable that the overall β-diversity and α-diversity were not significantly different following CFTR modulation with ivacaftor. This suggests that the alterations in gut microbiome in CF may also be affected by non-CFTR related factors, such as the high-calorie “CF diet”. While the life-prolonging benefits of the CF diet is irrefutable, a recent study has reported that many patients with CF are achieving their dietary targets through increased consumption of ‘junk foods’ high in energy and saturated fat^45^. Differences in bacterial profiles according to geographic location in our cohort supports the theory that ‘environmental’ factors, such as variations in CF care, and potential variations in diet and attitudes toward diet between Australia and Canada, influence the CF gut microbiome.

Our study has several limitations. The sample size was relatively small despite a multicenter approach and an extended follow-up period of a median of 6 months. This limits our study’s power and therefore our ability to do more extensive analysis and correlations with further clinical metadata. Furthermore, we may have missed any further alteration of the microbiome, which may occur with longer treatment time. Future validation studies based on a larger cohort and multiple sampling with dietary data over a longer period are needed. We also cannot determine from our current analysis whether any direct mechanisms of interaction exist or if our observations are due to indirect effects. To achieve such mechanistic insights, future work will require conducting experiments in *in vitro* CF models to evaluate the interactions between host (genetic, immune and mucosal) factors and microbial communities^46^.

In conclusion, ivacaftor therapy positively affects the gut microbiome as well as improves intestinal inflammation in individuals with CF carrying at least one gating *CFTR* mutation.

## Methods

### Patient cohort

Patients with CF carrying at least one gating CFTR mutation who were commencing treatment with ivacaftor at The Hospital for Sick Children (Toronto, CA), St Michael’s Hospital (Toronto, CA) and Sydney Children’s Hospital Randwick (Sydney, AU) were included in this study. Patients were excluded if they had a pre-existing gut disease.

### Stool and clinical data collection

Paired stool samples were collected from all patients before and after commencing ivacaftor therapy. Sample collection was were deferred/rejected if they had gastroenteritis or administered corticosteroids or non-steroidal ant-inflammatory drugs within two weeks of collection. All samples were stored at −20 °C until transfer to the laboratory, where they were then stored at −80 °C. All samples were processed together for microbial analysis. Clinical data including anthropometrics and FEV_1pp_ were collected at time of stool collection. Sweat chloride prior and after commencement of ivacaftor were also recorded.

### Stool inflammatory markers

Calprotectin was extracted and measured from frozen stool samples using the PhiCal kit (Calpro, San Diego, CA, US), according to manufacturer instructions. Fecal M2-PK was extracted and measured from stool samples using the ScheBo Tumour M2-PK test and protocol (ScheBo Biotech, Giessen, Germany). The lower limits of detection for the assays were calprotectin 5 mg/kg and M2-PK 1 U/mL. Normal values for faecal calprotectin and M2-PK were based on cut-offs of ≤50 mg/kg and ≤9U/ml respectively^47,48^.

### DNA extraction and genomic sequencing

DNA was extracted using the Thermo Fisher Scientific MagMAX Sample Preparation System (Waltham, MA). The 16S rRNA variable 3 (v3) gene region was amplified as previously described^49,50^. Amplification protocols were adapted from Whelan *et al*.^51^. Each sample reaction mixture contained 5pmol of each primer, 200 μM concentration of each deoxynucleoside triphosphate (dNTP), 1.5 mM MgCl_2_, and 1U *Taq* polymerase (Life Technologies, Carlsbad, CA). The PCR protocol consisted of an initial denaturation step at 95 °C for 5 minutes, 30 cycles, each step for 30 seconds, of 95 °C, 50 °C, and 72 °C, and a final extension step at 72 °C for 7 minutes. Previously described 341 F and 518 R 16s rRNA primers, modified for the Illumina platform (San Diego, CA) with 6-base pair unique barcode additions to the reverse primer, were utilized in this protocol^50^. Amplification of the v3 gene region was verified by electrophoresis on a 2% agarose gel. Amplicons were then sequenced using the Illumina MiSeq platform.

### Microbial composition and statistical analysis

Resulting paired-end sequences from Illumina sequencing are available on the Sequence Read Archive (SRP#162699). Sequences were processed using a custom in-house pipeline^51^. Briefly, this pipeline uses Cutadapt^52^ to trim any reads surpassing the length of the v3 region, PANDAseq^53^. to align the paired-end sequences, Sickle to stringently quality-filter sequences (minimum average quality of 30)^54^, QIIME to size filter for sequences between 100–250 base pairs and to check for and remove chimeras^55^, AbundantOTU+ (clustering threshold of 97%)^56^ to pick operational taxonomic units (OTUs), and Ribosomal Database Project classifier to assign taxonomy using Greengenes reference database (2011 release)^57,58^.

After removing singleton OTUs (those with only 1 read across all samples) and any OTU that did not have a bacterial taxonomic assignment, 3,426,702 total reads (average: 95,186 reads/sample; range: 43,688- 132,742) with 1683 total OTUs (average: 263 OTUs/sample; range: 131–443) remained. To measure β-diversity, samples were normalized using proportional abundance^59^ and the Bray-Curtis metric was used. To measure α-diversity, samples were rarefied to 40,000 reads. Differences in β-diversity were assessed with the adonis function in the vegan package. Identification of taxa changes associated treatment was conducted using the Analysis of Composition of Microbiomes (ANCOM)^26^,after filtering our dataset to remove OTUs with less than 10 reads. Remaining analysis was conducted on phyloseq^60^, and vegan^61^ packages on R^62^ and plotted using GraphPad Prism version 6.0 for Mac OS X (La Jolla, CA). Differences in α-diversity before and after therapy were assessed using paired t-tests.

### Ethics approval and consent to participate

The research ethics boards of the participating academic institutions approved the study (SickKids #1000036224, St. Michael’s Hospital #13-089, South Eastern Sydney Area Health Service #10/240), which was in compliance with the ethical principles outlined in the declaration of Helsinki. All participants, parents and/or legal guardian signed an informed consent prior to study enrolment.

## Electronic supplementary material

Supplemental Information

## Data Availability

Sequencing data are available on the Sequence Read Archive (SRP#162699).
