# Supplementary material for: Impact of CFTR modulation with Ivacaftor on Gut Microbiota and Intestinal Inflammation
Source: Sci Rep. 2018 Dec 13;8:17834. doi: 10.1038/s41598-018-36364-6 (PMC6292911; doi:10.1038/s41598-018-36364-6)
Supplement: Supplementary file 1 — Supplemental Information [file 41598_2018_36364_MOESM1_ESM.pdf]

Impact of CFTR modulation with Ivacaftor on Gut Microbiota and Intestinal Inflammation

Chee Y. Ooi[1,2,3 *#], Saad A. Syed[4,5,6#], Laura Rossi[4,5,6], Millie Garg[1], Bronwen Needham[1], Julie Avolio[7], Kelsey Young[7], Michael G. Surette[4,5,6], Tanja Gonska[7,8]

[1] School of Women's and Children's Health, Medicine, The University of New South Wales, Sydney, NSW, Australia

[2] Molecular and Integrative Cystic Fibrosis (miCF) Research Centre, Sydney Children's Hospital, Randwick, NSW, Australia

[3] Department of Gastroenterology, Sydney Children's Hospital, Randwick, NSW, Australia

[4]Department of Medicine, McMaster University, Hamilton, Ontario, Canada

[5]Department of Biochemistry & Biomedical Sciences, McMaster University, Hamilton, ON, Canada

[6]Farncombe Family Digestive Health Research Institute, McMaster University, Hamilton, ON, Canada

[7]Department of Paediatrics, Division of Gastroenterology, Hepatology and Nutrition, The Hospital for Sick Children, Toronto, Ontario, Canada

[8]Translational Medicine, Research Institute, The Hospital for Sick Children, Toronto, Ontario, Canada

[*]Corresponding author

[#]Co-first authors

SUPPLEMENTAL FIGURE LEGENDS

Figure S1: Gut microbiome communities do not appear different with taxonomic summaries (A) by treatment (B) or sex (C), and do not differ in species richness (Observed Species) (D) or evenness (Shannon Diversity) (E) following treatment.

Figure S2: Gut microbiome weakly cluster by geographic location (Sydney, Australia vs. Toronto, Canada).

Figure S1

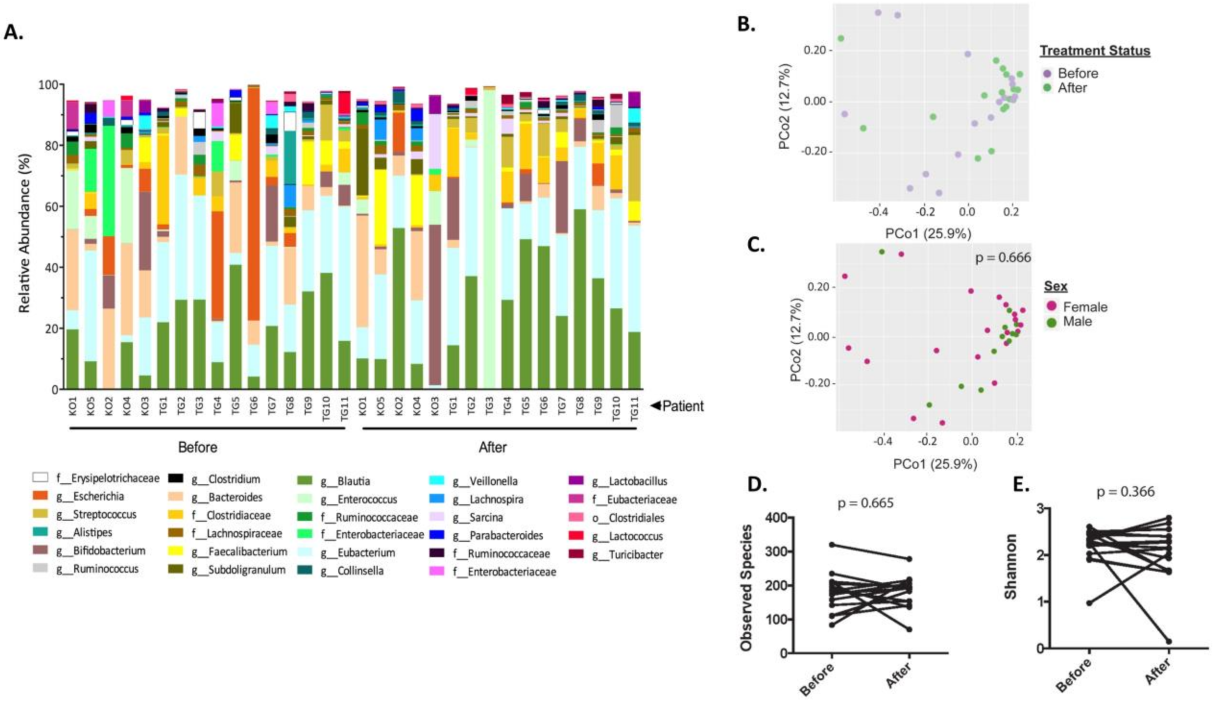

A.
Relative Abundance (%)
Patient
Before
After
f__Erysipelotrichaceae
g__Escherichia
g__Streptococcus
g__Alistipes
g__Bifidobacterium
g__Ruminococcus
g__Clostridium
g__Bacteroides
f__Clostridiaceae
f__Lachnospiraceae
g__Faecalibacterium
g__Subdoligranulum
g__Blautia
g__Enterococcus
f__Ruminococcaceae
f__Enterobacteriaceae
g__Eubacterium
g__Collinsella
g__Veillonella
g__Lachnospira
g__Sarcina
g__Parabacteroides
f__Ruminococcaceae
f__Enterobacteriaceae
g__Lactobacillus
f__Eubacteriaceae
o__Clostridiales
g__Lactococcus
g__Turicibacter
B.
Treatment Status
Before
After
PCo1 (25.9%)
PCo2 (12.7%)
C.
p = 0.666
Sex
Female
Male
PCo1 (25.9%)
PCo2 (12.7%)
D.
p = 0.665
Observed Species
Before
After
E.
p = 0.366
Shannon
Before
After

Figure S2

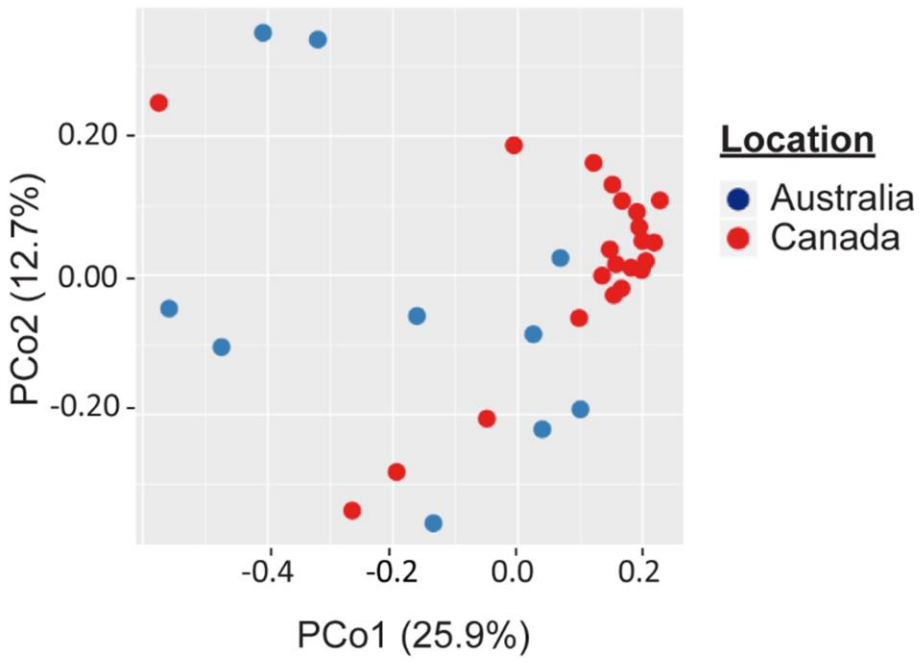

Location
Australia
Canada
0.20
0.00
-0.20
PCo2 (12.7%)
-0.4
-0.2
0.0
0.2
PCo1 (25.9%)
